# Preventing burnout: Community health worker perspectives on professional development and emotional labor overload - A qualitative descriptive study

**DOI:** 10.1371/journal.pone.0346430

**Published:** 2026-04-02

**Authors:** Meshack Achore, Martine Hackett, Tatiana Ramirez

**Affiliations:** Department of Population Health, School of Health Sciences, Hofstra University, Hempstead, New York, United States of America; The Chinese University of Hong Kong, HONG KONG

## Abstract

**Introduction:**

Community health workers (CHWs) play a critical role in advancing health equity by bridging underserved communities with health and social services. However, expanding responsibilities, emotional labor, and fragmented service systems contribute to burnout. While prior research has examined CHW burnout in urban, rural, and crisis contexts, little is known about how burnout is experienced and mitigated in suburban settings characterized by geographic dispersion and administrative fragmentation. This study explored how CHWs in suburban Long Island, New York, experience emotional labor overload and the strategies they use, individually and organizationally, to prevent burnout.

**Methods:**

We conducted a qualitative descriptive study using purposive and snowball sampling and recruited 10 CHWs from Nassau and Suffolk counties in New York. Data were collected through ten semi-structured interviews and five participant observations. Participants were primarily women aged 35–44 years, most of whom (86%) worked in Nassau County and reported 2–5 years of experience. Transcripts were analyzed thematically using Braun and Clarke’s six-step framework in ATLAS.ti.

**Results:**

Two overarching themes emerged: (1) Creating a healthy work-life balance, in which CHWs managed burnout by setting boundaries and practicing self-care; and (2) Building support structures in the workplace, where organizational supports such as supervision, peer collaboration, and ongoing training promoted resilience. Participants emphasized the importance of self-care rituals, spiritual grounding, and collegial networks as protective factors against emotional exhaustion.

**Conclusions:**

Burnout among suburban CHWs is shaped by the interaction of individual coping practices and organizational conditions within fragmented service systems. Strengthening supervision, peer support infrastructure, and professional development opportunities may enhance workforce resilience and sustainability. Investments in organizational support structures are critical to sustaining CHWs’ capacity to promote health equity.

## Introduction

Community health workers (CHWs) are frontline public health workers who are trusted members of, or have a deep understanding of, the communities they serve [1]. Through their cultural and linguistic knowledge, CHWs support individuals in accessing health and social services, provide health education, facilitate care navigation, and advocate for community needs [1–3]. By serving as bridges between health systems and underserved populations, CHWs play a critical role in advancing health equity and reducing disparities, particularly among marginalized communities in the United States [4,5].

The CHW workforce has expanded substantially over the past decade. Between 2012 and 2023, the number of CHWs in the United States increased by approximately 67%, from 38,020–63,400 workers, with continued growth projected over the next decade [6,7]. The workforce is predominantly female and racially and ethnically diverse, with most CHWs employed outside of hospital-based settings [8]. In New York State, recent Medicaid redesign initiatives and social care integration efforts, including the 1115 Waiver Amendment, have further expanded CHW roles and responsibilities, particularly in underserved suburban regions. While these initiatives underscore the value of CHWs, they also risk exacerbating emotional strain if workforce development and organizational supports are insufficient.

Despite their central role, CHWs often perform emotionally demanding work under conditions of limited resources, workforce shortages, and administrative burden. The relational nature of CHW work requires sustained emotional engagement with clients facing complex health and social challenges, including poverty, housing insecurity, chronic illness, and trauma. This emotional labor, frequently uncompensated and insufficiently recognized, places CHWs at heightened risk of burnout, a work-related syndrome characterized by emotional exhaustion, depersonalization, and reduced professional accomplishment [9–12].

Although burnout among CHWs has received increasing attention, important gaps remain in the literature. Existing studies have largely focused on urban safety-net systems, rural global health contexts, or crisis periods such as the COVID-19 pandemic, often emphasizing burnout prevalence or individual coping strategies. Far less is known about how CHWs experience and mitigate burnout in suburban settings, where structural inequities coexist with fragmented service systems and limited institutional visibility. Moreover, few qualitative studies have explicitly examined how professional development opportunities, supervision, and workplace support structures shape emotional labor and resilience among CHWs. As a result, burnout is frequently framed as an individual-level challenge rather than a relational and organizational phenomenon embedded within specific place-based contexts.

Suburban Long Island represents a theoretically and empirically important setting for examining CHW burnout. Unlike urban areas with dense healthcare infrastructures or rural communities characterized by tightly networked social systems, Long Island’s suburban counties are marked by persistent racial and economic segregation, geographically dispersed populations, and fragmented health and social service delivery. Nassau and Suffolk Counties exhibit pronounced health inequities, particularly among Black and Hispanic residents, alongside complex administrative systems that require clients to navigate multiple agencies, intake procedures, and eligibility requirements. In this context, CHWs serve as critical intermediaries, shouldering substantial emotional and logistical responsibilities while often operating without the organizational recognition or support afforded to more visible urban-based programs. These structural dynamics intensify emotional labor and heighten vulnerability to burnout, making suburban Long Island an underexplored but crucial site for understanding CHW workforce sustainability.

The purpose of this study is to examine how CHWs in suburban Long Island experience emotional labor overload and prevent burnout. Guided by Maslach’s multidimensional burnout framework, which conceptualizes burnout as emotional exhaustion, depersonalization, and reduced personal accomplishment [13], this study examines community health workers’ perspectives on emotional labor overload and the role of professional development and other coping strategies in burnout prevention. We examine how individual strategies (e.g., boundary setting and self-care) and organizational supports (e.g., supervision, peer collaboration, and professional development) interact to shape CHWs’ experiences of emotional labor and resilience. By focusing on CHWs in suburban Long Island, this study advances qualitative CHW burnout research in three key ways: first, by centering a suburban context that remains largely absent from existing scholarship; second, by framing burnout as a relational and organizational process rather than solely an individual condition; and third, by highlighting professional development and workplace support structures as critical, yet underexamined, mechanisms for burnout mitigation. Findings from this study have direct implications for CHW workforce development, organizational practice, and policy efforts aimed at strengthening health equity through sustainable community-based care.

## Methods

### Study setting

Long Island, a suburb of New York State, faces persistent racial inequities in healthcare access, treatment, and health outcomes. The Nassau and Suffolk counties have a long history of neglecting the health concerns of their Black residents due to structural racism that enables racially segregated housing, school districts, and physical environments. Black and Hispanic residents experience worse outcomes on many health indicators, highlighting the longstanding racial inequality in the area. The NYS Department of Health (2024) reports that 55.4% of Hispanic, 51.2% of Black, and 29.0% of White Nassau County residents die prematurely before age 75. The Black infant death rate in Nassau County is 5.5 per 1000 live births, while the rate for White infants is 2.0 per 1000 live births [14]. Additionally, Nassau and Suffolk Counties had 346,488 and 416,371 Medicaid beneficiaries in 2023, respectively [14]. Long Island accounts for 10% of New York’s Medicaid recipients [14]. Social care integrated with primary care has the potential to improve health outcomes for nearly 763,000 Long Islanders, or 27% of the population. Long Island’s human service system consists of hundreds of standalone agencies with varying capacities and intake procedures. To access the services they need, clients must navigate multiple agencies, intake processes, applications, and follow-ups within this fragmented social care system. New York State’s 1115 Waiver Amendment [15] aims to integrate primary care with a comprehensive social care network and a workforce development program to address health-related social needs. CHWs play a vital role in this initiative, especially on Long Island. By examining burnout among CHWs working in a diverse suburban area, our findings can help organizations build a strong CHW workforce, which is crucial for advancing health equity, empowering communities, and strengthening the U.S. public health system. The study was conducted in Nassau and Suffolk Counties, suburban regions of Long Island, New York. Providing contextual description aligns with qualitative descriptive methodology, which emphasizes situating findings within real-world practice settings to enhance transferability [16].

### Study design

The interview data were collected between April 2024 and September 2024, using a qualitative descriptive research design, an approach appropriate for research seeking a comprehensive, minimally theorized account of participants’ experiences in everyday language [16,17]. Semi-structured in-depth interviews and participant observations were conducted to explore experiences of professional development and emotional labor among community health workers (CHWs) serving clients in Nassau and Suffolk Counties, New York. A qualitative approach was used to capture how CHWs interpret their work experiences, construct meaning, and navigate their work environments. Semi-structured interviews allowed for consistency across participants while retaining flexibility, and participant observations provided insight into CHW work in naturalistic settings. The use of multiple data sources supported analytic triangulation.

### Participant eligibility

Eligible participants were current community health workers (CHWs), defined as frontline staff or paraprofessionals engaged in outreach, health education, care navigation, or community-based support. Inclusion criteria were: (1) active employment or engagement as a CHW in Nassau or Suffolk County; (2) age ≥ 18 years; (3) ability to communicate in English or Spanish (interviews were conducted in English, with occasional Spanish used by bilingual participants); (4) willingness to provide informed consent and participate in a 45–90-minute interview; and (5) at least six months of CHW experience. Eligibility criteria were defined to ensure participants had direct experiential knowledge of the phenomenon under study. In qualitative descriptive research, purposive inclusion of participants with practical experience is essential for obtaining information-rich accounts [18]. Exclusion criteria included supervisory-only roles without direct CHW duties, volunteers or interns with less than six months of experience, staff with exclusively administrative or clinical roles, and the inability to provide informed consent.

### Recruitment strategy

Purposive and snowball sampling were used to recruit CHWs with diverse experiences across counties and service settings. Qualitative descriptive studies often use purposive sampling to ensure variation while focusing on participants who can provide detailed descriptions of the phenomenon [17,18]. Purposive sampling was employed to ensure variation in gender, age, years of experience, primary county of work, and language use. Snowball sampling helped reach CHWs across different community organizations. Initial participants were identified through partner organizations and CHW networks and were invited via email or phone. Participants were also asked to recommend eligible peers. Recruitment continued until enough information was gathered. A brief demographic questionnaire was administered after each interview to record sample characteristics.

### Data collection

Data were collected through semi-structured interviews (n = 10) and non-participant observations (n = 5). Semi-structured interviews align with qualitative descriptive designs because they allow participants to describe experiences in their own words while covering core topics (17). The interviews were conducted via Zoom, lasted approximately 45–90 minutes, and were audio-recorded with participant consent. In addition to interviews, five observation sessions were conducted to contextualize the interview data and enrich the descriptive depth. Combining interviews with observations enhances descriptive validity and supports triangulation in qualitative research [18]. Observations took place over 2–4-hour periods in naturalistic CHW work settings, including community-based organizations, outreach events, team meetings, and case conferences. The main focus of the observations was on CHWs’ daily routines, their interpersonal interactions with clients and colleagues, emotional expressions during service delivery, boundary-setting practices, and the use of organizational supports such as supervision and peer consultation. Interviews were conducted via Zoom to accommodate participants’ schedules and geographic locations. While virtual interviews increased accessibility and comfort for participants, we recognize that this format may limit some contextual and embodied cues present in in-person interactions. To address this, interviewers paid close attention to vocal tone, pauses, and emotional expressions and supplemented interview data with detailed field notes and participant observations made in work settings.

Observations were non-participant and unobtrusive. Detailed field notes were recorded during and immediately after each observation, capturing contextual details, interactional dynamics, and reflexive insights related to emotional labor and workplace support. Following interviews, participants completed a brief demographic survey.

Maslach’s multidimensional burnout framework guided the development of the interview guide. Following the guidelines outlined by Kallio et al. [19], questions were intentionally crafted to explore experiences of emotional exhaustion (e.g., emotional demands and fatigue), depersonalization (e.g., emotional distancing or strain in client relationships), and professional accomplishment (e.g., perceived impact and meaning of CHW work), while giving participants flexibility to introduce unexpected themes.

### Sample size justification and saturation

The sample size was determined based on the principle of information power, which suggests that the more relevant information participants provide regarding a specific study goal, the fewer participants are needed [20]. Given the precise aim, the relatively homogeneous professional group, and the use of an established theoretical framework, a modest sample size was suitable. Empirical research shows that code saturation often occurs after about 9 interviews in focused qualitative studies [21], with systematic reviews supporting small samples once analytical depth is reached [22]. After the tenth interview and fifth observation, no new substantive codes or themes appeared, and further analysis confirmed stability in the coding framework, indicating thematic saturation.

### Ethical considerations

Ethical approval was obtained from Hofstra University’s Institutional Review Board (Reference number: 20240320-PH-HPHS-ACH-1). Written informed consent was obtained from all interview participants. For participant observations, verbal and/or written consent was obtained from CHWs and organizational supervisors prior to observation. When observations occurred in group settings (e.g., team meetings or case conferences), all individuals present were informed of the researcher’s role and the purpose of the observation. No identifying information about clients or non-consenting individuals was recorded. The study adhered to the Declaration of Helsinki.

### Reflexivity

Reflexivity was maintained throughout the research process. The authors acknowledged their positionality as public health researchers with both insider familiarity and potential power asymmetries. Reflexive journaling was used after interviews to document assumptions, emerging insights, and potential biases, which were revisited during team analysis meetings to support interpretive transparency. This is consistent with recommendations that qualitative researchers explicitly articulate methodological positioning and analytic decisions [18].

### Data analysis

Audio recordings were transcribed verbatim using an AI-powered transcription service and reviewed for accuracy by two research assistants. All transcripts and field notes were de-identified prior to analysis. Data were analyzed thematically using Braun and Clarke’s [23] six-phase framework and managed with ATLAS.ti (version 18). An initial codebook was developed inductively from early transcripts and refined iteratively through team discussion. All transcripts were independently coded by the three authors, with consensus reached through negotiated agreement. Discrepant cases were retained to refine conceptual boundaries. Observation field notes were coded using the final codebook and integrated into theme development to support triangulation. All transcripts were independently coded by three authors. Coding discrepancies were discussed in analytic meetings and resolved through negotiated consensus. This collaborative process minimized premature closure and deepened interpretation [24].

Although coding was inductive, Maslach’s burnout framework was applied deductively during later stages of analysis to support interpretation. Higher-order themes were examined in relation to emotional exhaustion, depersonalization, and professional accomplishment. This analytic strategy reflects qualitative description in integrating existing conceptual frameworks while maintaining descriptive fidelity [17].

The study adhered to the Consolidated Criteria for Reporting Qualitative Research (COREQ), thereby ensuring transparency across the study design, participant selection, reflexivity, data collection, and analysis [20].

### Rigor and trustworthiness

Trustworthiness was established using Lincoln and Guba’s [25] criteria of credibility, transferability, dependability, confirmability, and authenticity, as well as contemporary guidance on methodological coherence [26]. Credibility was improved through triangulation of interviews and observations, independent coding by multiple researchers, and negotiated consensus during analysis meetings. Discrepant cases were kept, refining the thematic boundaries. Transferability was supported through detailed descriptions of the suburban context, participant characteristics, and organizational settings. Dependability and confirmability were strengthened by maintaining an audit trail of coding decisions and codebook updates, as well as through reflexive journaling to reveal assumptions and reduce bias. Findings were grounded in verbatim quotations to ensure transparency in analysis. Authenticity was supported by depicting both shared and differing participant experiences.

## Results

### Participant demographics

Ten (10) community health workers (CHWs) participated in this study, representing diverse experiences and client populations across Nassau and Suffolk Counties, New York. Participants were predominantly women (approximately two-thirds) and ranged in age from their late twenties to mid-forties, with most (86%) between 35 and 44 years. The majority (85.8%) had between two and five years of experience as CHWs, and nearly all (86%) worked primarily in Nassau County, serving low-income families, mothers, older adults, and Medicare beneficiaries. Most participants were bilingual, with Spanish being the most frequently used language other than English. All participants reported working within their communities of residence, underscoring the relational proximity between CHWs and their clients. Three participants did not complete the post-interview demographic questionnaire due to time or technical limitations; however, their narratives were included in the thematic analysis, which focused on the full set of qualitative data.

Although ten CHWs participated in interviews, only seven completed the brief post-interview demographic survey (Table 1). The remaining three participants declined to complete the form due to time constraints immediately after their interviews or technical difficulties with the electronic link. All ten interviews, however, were fully recorded and transcribed, providing rich qualitative data for analysis. The missing demographic information did not affect the integrity of the findings because thematic saturation was achieved across interviews, and themes were derived from the full qualitative dataset rather than from demographic variables. Demographic summaries were used only for descriptive context, not as analytic strata influencing coding or theme generation.

**Table 1:** Demographic characteristics of study participants.

Characteristics		n	%
**Years of experience**	2-5 years	**6**	**85.7**
	15 + years	**1**	**14.3**
**Age**	35-44 years	**1**	**14.3**
	45-54 years	**6**	**85.7**
**Gender**	Female	**5**	**71.4**
	Male	**2**	**28.6**
**Race/Ethnicity**	Black or African American	**1**	**14.3**
	Hispanic	**4**	**57.1**
	Asian	**2**	**28.6**
**Additional Languages used**	Spanish	**4**	**57.1**
	Haitian Creole	**1**	**14.3**
	French	**1**	**14.3**
	Tamil	**1**	**14.3**
	Telugu	**1**	**14.3**
**Educational Level**	Some college	**2**	**28.6**
	2-years degree	**2**	**28.6**
	4-year degree	**1**	**14.3**
	Professional Degree	**2**	**28.6**
**Service Area**	Nassau county	**6**	**85.7**
	Both Nassau and Suffolk	**1**	**14.3**
**Work Characteristics**	Full time employee	**7**	**100**
	Has an additional job	**7**	**100**
	Member of community served	**6**	**85.7**
	Conducts home visits	**3**	**42.9**
	Received initial training	**6**	**85.7**
	Participates in continuing education	**7**	**100**

### Overview of themes and sub-themes and coding structure

Analysis followed Braun and Clarke’s six-phase framework. Initial open coding generated 132 codes, which were iteratively grouped into six subthemes and two overarching themes through team-based analysis. Subthemes represent patterned responses across participants, while themes capture higher-order organizing concepts grounded in participants’ accounts (Table 2). When interpreted through Maslach’s burnout framework, Theme 1 primarily reflects strategies to address emotional exhaustion, whereas Theme 2 captures organizational resources that mitigate depersonalization and reinforce professional accomplishment. Participant observations corroborated interview findings by documenting real-time boundary-setting practices, informal peer support exchanges, and supervisory interactions, and in some cases, revealed emotional strain and task-sharing behaviors that participants normalized or minimized in interviews.

**Table 2:** Identified interview themes and sub-themes.

Theme	Sub-themes
Creating a Healthy Work-Life Balance	Establishing Boundaries
	Self-Care
Support Structures in the Workplace	Case Conferences Provide Support
	Active Supervision
	Coworkers Support Each Other
	Additional Educational Opportunities

- **Theme 1:** Creating a Healthy Work–Life Balance (individual strategies)
  - Subtheme 1.1: Establishing boundaries
  - Subtheme 1.2: Practicing self-care
- **Theme 2:** Building Support Structures in the Workplace (organizational strategies)
  - Subtheme 2.1: Case conferences provide support
  - Subtheme 2.2: Active supervision
  - Subtheme 2.3: Coworker collaboration and task-sharing
  - Subtheme 2.4: Access to additional educational opportunities

To enhance analytic transparency, the process of theme development followed an inductive–deductive approach. Initial open codes were generated line by line from each transcript to capture participants’ language and meanings. Similar codes were clustered into categories that represented broader patterns across interviews (e.g., “emotional depletion,” “work–home spillover,” “peer learning,” “training access”). These categories were then compared and refined through iterative team discussions to identify subthemes, defined as coherent clusters of related codes that reflected distinct aspects of the participants’ coping strategies. Subthemes were then organized under higher-order themes that captured the overall conceptual structure of burnout prevention among CHWs. Field notes and observation data were used to confirm or elaborate subthemes, ensuring analytic triangulation. A reflexive audit trail documenting coding decisions and revisions was maintained throughout analysis.

### Theme 1: Creating a healthy work–life balance

Participants consistently described burnout as emerging when emotional demands exceeded their capacity to disengage from clients’ needs. In response, CHWs developed intentional strategies to regulate emotional labor, primarily through boundary-setting and self-care. These practices were framed not as personal preferences but as professional necessities learned over time.

#### Subtheme 1.1: Establishing boundaries.

Boundary-setting emerged as a critical professional skill that evolved through experience. Early-career CHWs described difficulty limiting availability due to feelings of responsibility and guilt toward clients, often leading to emotional depletion. Over time, participants reframed boundaries as essential to sustaining their ability to provide care.

One participant reflected on this learning process:

“At first, I felt bad not answering late-night calls because clients depend on me. But I realized I can’t pour from an empty cup.” (Participant 4, 2 years’ experience, Nassau County)

More experienced CHWs emphasized firm temporal boundaries as protective mechanisms, particularly disengaging from work outside scheduled hours:

“When I leave work, I leave work. I don’t carry my phone with me. When I’m off, I’m off.” (Participant 7, 5 years’ experience, Suffolk County)

Another participant highlighted the psychological impact of emotionally intense disclosures, underscoring why boundaries were necessary:

“It’s impossible for clients to disclose difficult experiences to you without it affecting you psychologically.” (Participant 2, 3 years’ experience, Nassau County)

Together, these accounts illustrate boundary-setting as an adaptive response to emotional exhaustion that enables sustained engagement rather than disengagement from clients.

#### Subtheme 1.2: Practicing self-care.

Self-care was described as a deliberate strategy for emotional regulation rather than an optional wellness activity. Participants emphasized holistic practices that addressed physical, emotional, and spiritual well-being, often rooted in cultural or faith-based traditions. These practices were framed as essential for maintaining emotional balance in demanding work environments.

One participant described self-care as a way of restoring personal identity beyond work:

“Whenever I feel overwhelmed, I go to the spa or get my nails done, whatever makes me feel human again.” (Participant 1, 4 years’ experience, Nassau County)

Others emphasized spiritual grounding as central to emotional resilience:

“I exercise, eat well, and pray. Staying connected with God keeps me grounded.” (Participant 9, 5 years’ experience, Suffolk County)

Another participant described self-care as an integrated, ongoing practice:

“I work on my mind by maintaining mental stability. I pray often and stay connected with God. I believe grace and mercy are what keep everything balanced.” (Participant 4, 2 years’ experience, Nassau County)

Analytically, self-care functioned as a compensatory strategy that allowed CHWs to manage emotional labor in contexts where structural supports were insufficient or inconsistent.

### Theme 2: Building support structures in the workplace

While individual strategies were critical, participants consistently emphasized that burnout mitigation depended on organizational conditions that distributed emotional labor and normalized support-seeking. Workplace structures shaped how CHWs processed emotionally complex cases, navigated ethical dilemmas, and sustained professional motivation.

#### Subtheme 2.1: Case conferences provide support.

Structured case conferences emerged as collective spaces for emotional processing and problem-solving. Participants described these forums as reducing isolation by allowing CHWs to share responsibility for complex cases.

One participant explained the structure and impact of these meetings:

“Every day, a different CHW would talk about a client they thought might present challenges. Then we would work together as a team to brainstorm solutions… I don’t have to go through this alone.” (Participant 3, 4 years’ experience, Nassau County)

Another emphasized the emotional normalization that occurred during case discussions:

“If someone had too much going on, we supported each other to come up with solutions.” (Participant 3, 4 years’ experience, Nassau County)

Observational data confirmed that case conferences functioned as both technical and emotional support mechanisms, reinforcing collective responsibility and reducing emotional burden.

#### Subtheme 2.2: Active supervision.

Supportive supervision emerged as a key organizational buffer against burnout. Participants described supervisors as accessible and emotionally attuned, providing guidance not only on tasks but also on managing emotional strain.

One participant highlighted the role of supervision in emotional regulation:

“When I talked with my supervisor, it was a chance to process things and practice mindfulness. She checks in to make sure I’m not letting clients drain me.” (Participant 6, 2 years’ experience, Nassau County)

Another emphasized supervisors’ role during difficult encounters:

“After a bad encounter, she’ll say, ‘Do A, B, C, D, and come back when you feel okay.’ She’s very supportive.” (Participant 10, 5 years’ experience, Suffolk County)

Analytically, supervision functioned as emotional containment, reinforcing professional confidence and reducing depersonalization.

#### Subtheme 2.3: Coworker collaboration and task-sharing.

Informal peer support was a routine feature of CHW work and played a critical role in managing workload and emotional strain. Participants described frequent collaboration, information-sharing, and task redistribution as strategies to prevent overload.

One participant described peer learning and shared problem-solving:

“We get feedback from each other and learn from each other. We share experiences and talk through how to handle situations.” (Participant 7, 5 years’ experience, Suffolk County)

Another highlighted task-sharing as a way to prevent burnout:

“If someone has two births or postpartum situations, they’ll ask me to step in so it doesn’t become overwhelming.” (Participant 10, 5 years’ experience, Suffolk County)

Observations revealed that these exchanges were frequent and informal, indicating that peer support was embedded in everyday practice rather than activated only during crises.

#### Subtheme 2.4: Access to additional educational opportunities.

Participants described ongoing training as both skill-enhancing and emotionally validating. Professional development opportunities were viewed as recognition of the expertise required for CHW work and as mechanisms for sustaining motivation and career advancement.

One participant emphasized the practical value of training:

“If someone is well-trained, they can help families handle situations like Alzheimer’s more effectively.” (Participant 8, 16 years’ experience, Suffolk County)

Another described training as contributing to long-term career development:

“The training was enriching. Some of us stayed as CHWs, others moved into different roles, and those certifications helped us.” (Participant 10, 5 years’ experience, Suffolk County)

Participants emphasized that ongoing, rather than one-time, training was necessary to maintain competence and engagement, reinforcing professional accomplishment.

## Discussion

This study explored how community health workers (CHWs) in suburban Long Island experience burnout and how they mitigate it through both individual and organizational strategies. In line with Maslach's multidimensional framework of burnout, which defines emotional exhaustion, depersonalization, and diminished personal accomplishment as interconnected consequences of chronic work stress [27], the results indicate that CHWs manage burnout through a dynamic interaction of personal agency and institutional support. Individual strategies, such as setting clear boundaries and engaging in self-care, directly addressed emotional exhaustion by allowing CHWs to regulate the intensity of their work engagement. As one participant shared, *“When I leave work, I leave work. I don’t carry my phone with me… when I’m off, I’m off.”* In contrast, organizational mechanisms such as supervision, peer collaboration, and access to professional development functioned as structural buffers that reduced depersonalization and reinforced a sense of professional accomplishment [27,28].

Suburban settings are often assumed to be resource-rich; however, this study’s findings demonstrate that suburban fragmentation produces distinct forms of emotional labor and burnout among community health workers (CHWs). Unlike urban contexts with dense, co-located service infrastructures or rural settings where resource scarcity is visible and often collectively navigated, suburban systems are characterized by geographic dispersion, administrative decentralization, and weak inter-organizational coordination. In this environment, CHWs frequently operate across multiple disconnected agencies and municipalities, functioning as informal system integrators rather than solely as frontline providers. Geographic dispersion increases travel time, limits informal peer interaction, and reduces opportunities for shared emotional processing, resulting in greater professional isolation and intensified emotional labor that is absorbed at the individual level. Service silos further amplify burnout by obscuring organizational accountability and increasing role ambiguity. CHWs in suburban contexts often bear responsibility for navigating fragmented health and social care systems without the authority or institutional support needed to resolve structural barriers. This intermediary role heightens emotional exhaustion and moral strain, as CHWs witness unmet client needs while lacking the capacity to effect system-level change. Viewed through Maslach’s multidimensional burnout framework, suburban fragmentation concentrates emotional labor in ways that exacerbate emotional exhaustion, foster depersonalization through isolation and role overload, and undermine professional accomplishment by limiting CHWs’ ability to translate sustained effort into visible impact. Together, these findings position suburban CHW burnout as a structurally mediated, place-specific phenomenon, underscoring the need for workforce strategies and policies that address coordination failures and institutional invisibility rather than relying solely on individual coping or resilience.

Concern about CHW burnout, defined as a work-related stress syndrome caused by prolonged exposure to poorly managed job stress [12], has increased globally. In New York State, burnout is especially concerning because CHWs face an increased workload due to new initiatives aimed at promoting health equity, Medicaid redesign, and community-based public health care. This study investigates burnout among CHWs on Long Island, a suburban region in New York State, and explores the strategies they have developed to manage work-related stress. In line with the definition of burnout, participants report feeling stressed, emotionally isolated, and exhausted when they are unable to disconnect from work after hours and lack training in setting work boundaries. These findings align with those of other scholars [29,30]. For example, De Hert [29] found in his review that healthcare workers faced an increased risk of burnout, including higher levels of mental distress and poor sleep quality during the COVID-19 pandemic. Participants described persistent emotional strain, exhaustion, and psychological burden associated with their work. While these experiences overlap conceptually with symptoms often discussed in mental health research, this study did not assess clinical anxiety or depression. Rather, the findings reflect participants’ subjective experiences of emotional stress within their work contexts. Factors contributing to these mental strains include poor working conditions, unclear role definitions, and uncertainty about when and how to disconnect from work.

We found that the mental health symptoms and emotional distress experienced by the CHWs because of their work drove them to develop strategies to cope, manage, or reduce burnout and work overload. These strategies vary and include both personal and organizational interventions. One personal-level intervention used by CHWs to prevent burnout was *setting boundaries* with their clients. Participants refused to take calls after their shifts, turned off their phones once they left work, and set clear expectations with their clients about their availability. These findings align with those of Ballard et al. [31], who found that clear role delineation is a key protective strategy used by CHWs and other frontline workers to prevent overload and burnout. Similarly, in her research, “Burnout: A multidimensional perspective,” Maslach [27] argued that role clarity and boundary setting can serve as buffers against CHW burnout. Participants also highlighted the benefits of taking control of their personal time on their psychological and emotional well-being. This supports previous findings that greater “work-time control,” or an employee’s autonomy regarding work hours, was significantly associated with less internal work-to-home interference, defined as a “psychological preoccupation with one domain of life (e.g., work) while within the role boundaries of another domain of life (e.g., family)” [32]. The same study also found that greater internal work-to-home interference was significantly associated with increased exhaustion, a warning sign of burnout [32]. The current study reinforces prior research indicating that work-time control can play an essential role in reducing CHWs’ tendency to ruminate about their work and, consequently, preventing burnout.

Another personal-level coping strategy that emerged from our analysis was self-care. Self-care for our study participants took various forms, including spiritual practices, family support, exercise, and beauty rituals. These self-care practices, as emphasized by the study participants, are not optional; they are essential and integral to maintaining energy and building resilience against burnout. This finding aligns with other research. A related study by Ndulue et al. [33] found that self-care strategies, such as regular yoga and sharing with family members, helped CHWs overcome challenges they faced in their work. Additionally, several participants in the current study identified prayer and faith as grounding influences, highlighting how deeply spiritualized self-care is. Chisango et al. [34] also observed this tendency in studies of Black CHWs. Iosim et al. [35] have shown associations between a lack of spirituality and an increased risk of burnout among workers in the helping professions. CHWs’ holistic approach to wellness (i.e., mental, physical, and spiritual) reflects a culturally rooted paradigm of care and coping that is sometimes overlooked in the development of the broader health profession.

The current findings emphasize the importance of supportive organizational structures as buffers against burnout, particularly case conferences, active supervision, and a collaborative team culture. Participants view these supports as opportunities for technical guidance, emotional processing, and affirmation. These results align with studies by Boustani et al. [36], who found that CHWs working in programs that offered peer learning opportunities, emotional support, and regular supervision experienced substantially lower rates of turnover and burnout. Case conferences can benefit healthcare workers who serve patients with complex social needs by reducing burnout, increasing job satisfaction, and raising awareness of available programs and services to support patients [37]. Additionally, other studies have shown that high-quality supervision can reduce the risk of burnout among workers in social service and healthcare roles [35,38]. Finally, colleague support has been shown to decrease burnout among healthcare workers [39,40]. The organizational structures highlighted in our study indicate the benefits of fostering community and interpersonal support among CHWs, which can reduce burnout risk and, in turn, improve client care.

A common theme among CHWs was their desire to pursue additional training and education opportunities for both professional development and their own client care. These opportunities can motivate CHWs to stay in this field. Previous research has also shown that additional training and education can help reduce burnout [41]. The chance to explore learning interests may be motivating and satisfying for CHWs. This finding provides a foundation for future research on how training, education, and professional development affect CHWs.

### Study recommendations

The findings of this study underscore that while individual coping strategies are important for mitigating emotional exhaustion, burnout among community health workers (CHWs) is fundamentally shaped by organizational and structural conditions. Accordingly, effective burnout prevention requires action across multiple levels, including individual practice, organizational accountability, and broader policy and funding structures.

### Individual-level strategies

CHWs described boundary-setting, self-care, and peer advocacy as important strategies for managing emotional strain. These practices, such as setting limits on availability, seeking peer support, and prioritizing personal well-being, can help CHWs regulate emotional labor and sustain engagement in demanding roles. However, these strategies should be understood as supportive coping mechanisms rather than substitutes for institutional responsibility. Expecting CHWs to manage burnout primarily through individual resilience risks normalizing structural inadequacies and obscuring the role of organizations and systems in producing emotional overload.

### Organizational-level obligations

Organizations employing CHWs bear primary responsibility for creating conditions that prevent burnout. The findings highlight the importance of active and emotionally attuned supervision, structured case conferences, and formal peer support mechanisms that redistribute emotional labor rather than individualize it. Organizations should institutionalize protected time for reflection and debriefing, establish clear role boundaries, and integrate self-care and boundary-setting into formal training and supervision rather than treating them as personal choices. Additionally, organizations should ensure equitable access to ongoing professional development and recognize emotional labor as a core component of CHW work within job descriptions and performance expectations.

### Structural and policy-level interventions

At the structural level, burnout prevention requires sustained investment in the CHW workforce and reforms that address fragmented service systems, particularly in suburban contexts. Policymakers and funders should prioritize stable, long-term funding streams that support adequate staffing, supervision, and training rather than short-term, grant-dependent models that exacerbate job insecurity and emotional strain. Workforce policies should explicitly fund supervisory roles, peer support infrastructure, and continuing education as essential components of CHW programs. In addition, efforts to improve inter-agency coordination and reduce service silos, such as shared referral platforms or integrated care networks, can alleviate the coordination burden currently absorbed by CHWs. Addressing burnout at this level shifts responsibility from individual workers to the systems that structure their labor.

### Study limitations

Several limitations should be acknowledged when interpreting the findings of this study. First, as a qualitative study relying on self-reported data, participants’ accounts may be influenced by social desirability or recall bias, despite efforts to build rapport and ensure confidentiality. Second, the use of purposive and snowball sampling may have resulted in a relatively homogeneous sample drawn from overlapping professional networks, potentially limiting the range of perspectives captured, particularly those of CHWs working in less supportive or more precarious organizational settings. Third, the heavy concentration of participants in Nassau County may have shaped the findings toward experiences specific to that service and organizational context, underrepresenting CHWs working in more rural or resource-constrained areas of Suffolk County. This study did not include standardized measures of mental health conditions; therefore, references to emotional distress reflect participants’ self-described experiences rather than clinically diagnosed anxiety or depression. Lastly, many participants were embedded in organizations that offered active supervision, peer collaboration, and professional development opportunities. While these contexts provided important insight into resilience-building practices, they may also have introduced a positive bias toward organizational support strategies, potentially amplifying their prominence in the findings.

Taken together, these factors underscore the context-specific nature of the results and suggest that the identified themes reflect how burnout is navigated within relatively supportive suburban CHW environments rather than a comprehensive depiction of all CHW working conditions. Nevertheless, triangulation through field observations and multi-coder analysis enhanced the credibility and trustworthiness of the results. Future research should incorporate larger and more socioeconomically diverse CHW samples and mixed-methods designs to validate and extend these findings.

## Conclusion

Overall, this study advances our understanding of burnout by showing how CHWs’ experiences bridge the micro (individual) and meso (organizational) dimensions of emotional labor and by demonstrating that addressing burnout requires structural, not just individual, solutions. Our findings carry clear implications for CHW workforce development: training curricula should explicitly integrate emotional resilience, boundary-setting, and self-care (for example, mindfulness, peer supervision, and stress-management modules) so emotional regulation is normalized as a professional competency; organizations should institutionalize active supervision, routine reflective practice sessions, and protected time for debriefing and collaborative problem-solving; and funders and health administrators should treat supervision and ongoing education as essential budgeted components of CHW programs, prioritizing trauma-informed care and cultural humility to reduce burnout and improve retention. As one participant summarized, “We take care of the community, but someone also has to take care of us.” Future research should test these recommendations across diverse geographic and institutional contexts, using mixed-methods designs to link burnout-mitigation strategies with measurable health and retention outcomes.
